# Awareness About Dry Eye Symptoms and Risk Factors Among Eastern Province Population in Saudi Arabia

**DOI:** 10.7759/cureus.48197

**Published:** 2023-11-03

**Authors:** Abdulaziz I AlSomali, Mohammed A Alsaad, Alya A Alshammary, Abdullah M Al-Omair, Raghad M Alqahtani, Ahmed S Almalki, Ali E Alhejji, Wafa Y Alqahtani

**Affiliations:** 1 Ophthalmology, King Faisal University, Al-Ahsa, SAU; 2 College of Medicine, King Faisal University, Al-Ahsa, SAU; 3 Ophthalmology, King Abdulaziz University Faculty of Medicine, Jeddah, SAU

**Keywords:** prevalence of dry eye symptoms, eastern province, dry eye, prevalence, saudi arabia, alahsa, ophthalmology

## Abstract

Background

Dry eye syndrome (DES), also known as keratoconjunctivitis sicca (KCS), is a common cause of patient's visits to the ophthalmologist. It is characterized by a defect in the tear film homeostasis, symptoms of ocular discomfort, and visual disturbance. Also, it increases the risk of ocular surface damage if complicated by tear film hyperosmolarity and ocular surface inflammation. The present study aims to measure awareness about dry eye disease and the risk factors among the Eastern region population in Saudi Arabia via an online questionnaire.

Methods

This is a cross-sectional community-based study conducted in Saudi Arabia that assessed knowledge and awareness of dry eye among the eastern province population using a self-administered online questionnaire. The minimum sample size was 385 adults. Statistical analysis was performed using SPSS software, version 21.0 (IBM Corp., Armonk, NY), and participants' overall awareness level was evaluated based on correct answers.

Results

In this study, a total of 522 participants fulfilling the inclusion criteria completed the study questionnaire. Participants ranged from 18 to 65 years, with a mean age of 27.2 ± 14.6 years old. Females represented a higher percentage of the sample, 341 (65.3%). Public awareness regarding dry eye diseases in the Eastern region, Saudi Arabia of 149 participants (28.5%) is overall a good awareness level while 373 (71.5%) of them had poor awareness.

Conclusion

Participants showed unsatisfactory results in awareness of risk factors of dry eye symptoms, where the most reported causes were prolonged concentration while using electronic devices, climatic factors, and lacrimal gland disorders.

## Introduction

Dry eye syndrome (DES), also known as keratoconjunctivitis sicca (KCS), is considered a common cause of patient visits to the ophthalmologist [1]. The International Dry Eye WorkShop (2007) defined DES as follows: Dry eye is a multifactorial disease characterized by a defect in the tear film homeostasis and symptoms of ocular discomfort and visual disturbance, with increased risk of ocular surface damage if complicated by tear film hyperosmolarity and ocular surface inflammation [2]. The tear film consists of three components: lipid produced by the Meibomian gland, aqueous produced by the lacrimal and accessory lacrimal glands, and mucin produced by goblet cells. Unbalance between those three layers can cause DES [1]. DES can negatively affect the productivity of workers, especially office workers [3]. In Saudi Arabia, DES is considered a highly prevalent and underdiagnosed disease [4-5]. In Jeddah, a study of a 251 normal population, the prevalence of DES was 234 (93.2%) [5]. On the other hand, in Al-Ahsa, a study of the 1858 population showed a prevalence of DES was 597 (32.1%) [6]. Furthermore, another study in Saudi Arabia noted a defect in the knowledge and habits regarding DES; however, there was a small number of participants in the eastern region (17, 3.8%) [7]. Considering the variability of the prevalence rate in Saudi Arabia and the lack of information about the knowledge of DES among the eastern province population, this study aims to establish reliable pieces of information regarding the awareness of dry eye syndrome among the eastern province population and to note whether we need to increase awareness in this region or not.

## Materials and methods

Methods

This is a descriptive community-based cross-sectional study based on a self-administer online questionnaire that evaluates the knowledge and awareness of dry eye among the eastern province population in Saudi Arabia. The data was collected by using a previously validated questionnaire by Khalid et al. [7]. The sample size was calculated using the formula n = z2pq\d2. With a confidence level of 95%, an estimated proportion of 50%, and a 5% level of precision, the minimum sample size was calculated to be 385. The actual sample size was 522 participants. Inclusion criteria included all adults aged 18 years and above of both genders and living in the eastern province. Those who were below 18 years of age and not living in the eastern province were excluded. An online, self-administered questionnaire was distributed through social media. Five sections were included in the questionnaire; the first is a consent form to join the research; the second is related to demographic data; the third contains questions about past medical history. The fourth section includes questions about caring for eyes, and the fifth section questions assessing the awareness and knowledge regarding DES.

Data analysis

The data were collected, reviewed, and then fed to Statistical Package for Social Sciences (SPSS), version 21.0 (IBM Corp., Armonk, NY). All statistical methods were two-tailed with an alpha level of 0.05, considering significance if the P value was less than or equal to 0.05. Regarding participant awareness, each correct answer was given a 1-point score. Overall awareness level regarding dry eye disease was assessed by summing up discrete scores for different correct awareness items. If the total score was 60% or more of the total possible score, the level of awareness was considered good, and scores less than 60% were considered poor. Descriptive analysis was done by prescribing frequency distribution and percentage for study variables, including participant data, behavior, and practice. Also, participants' awareness of (DES) was tabulated while the overall awareness level was graphed. Cross tabulation was used to show participants' overall awareness level distribution by their data and other factors using the Pearson Chi-square test for significance and exact probability test if there were small frequency distributions.

Ethical consideration 

After receiving participant consent to participate in the study, it will be conducted. The individual has the ability to withdraw at any point during the study as it is voluntary. No efforts are made to identify the subjects. The ethical clearance was given by the Deanship of Scientific Research at King Faisal University on September 13, 2022 (KFU-REC-2022-SEP-ETHICS166).

## Results

A total of 522 participants fulfilling the inclusion criteria completed the study questionnaire. Participant's ages ranged from 18 to 65 years, with a mean age of 27.2 ± 14.6 years old. 341 (65.3%) were females and 181 (34.7%) were males. As for job titles, 173 (33.1%) were in the governmental sector, 112 (21.5%) worked in the healthcare field, and 135 (25.9%) were not employed. A total of 36 (6.9%) participants complained of thyroid disorders, 26 (5%) had diabetes mellitus, 25 (4.8%) complained of hypercholesterolemia, and 382 (73.2%) had no chronic health problem. A total of 133 (25.5%) used medications, while 68 (13.0%) underwent refractive surgery, and 440 (84.3%) had no eye surgery (Table 1).

**Table 1:** Bio-demographic data of study participants in the Eastern region of Saudi Arabia DM: Diabetes Mellitus; HTN: Hypertension

Bio-demographic data	No	%
Age in years		
18-25	204	39.1%
26-35	117	22.4%
36-55	181	34.7%
> 55	20	3.8%
Gender		
Male	181	34.7%
Female	341	65.3%
Job title		
Not employed	135	25.9%
Student	41	7.9%
Health care field	112	21.5%
Governmental sector	173	33.1%
Private/free works	61	11.7%
Chronic diseases		
None	382	73.2%
DM	26	5.0%
HTN	22	4.2%
Thyroid disorders	36	6.9%
Hypercholesterolemia	25	4.8%
Asthma	23	4.4%
Others	36	6.9%
Use certain medications		
Yes	133	25.5%
No	389	74.5%
Have you had eye surgery?		
None	440	84.3%
Refractive error correction	68	13.0%
Corneal transplantation	3	0.6%
Cataract	5	1.0%
Others	6	1.1%

Table 2 showseye-related practices and behavior among study participants in the Eastern region of Saudi Arabia. Two hundred and one (38.5%) of the participants reported they wear medical glasses, 185 (35.4%) used sunglasses, 185 (35.4%) used eyedrops to moisturize their eyes, 73 (14.0%) used lenses, while 142 (27.2%) did not use any of these. Also, 316 (60.5%) participants rub their eyes, 312 (59.8%) use E-devices for a long time continuously, 181 (34.7%) use cosmetic eye tools, and 66 (12.6%) may direct the air into the eye. Among participants, only 63 (12.1%) visited a doctor regularly.

**Table 2:** Eye-related practice and behavior among study participants in the Eastern region of Saudi Arabia

Self-reported behaviour	N	%
Do you use any of the following?		
Medical glasses	201	38.5%
Sunglasses	185	35.4%
Eye drops to moisturize	185	35.4%
Lenses	73	14.0%
Ointments to moisturize the eyes	24	4.6%
None of these	142	27.2%
Do you engage in any of the following behaviors?		
Rubbing the eye	316	60.5%
Long time using the devices without a break	312	59.8%
Cosmetic eye tools	181	34.7%
Direct the air into the eye	66	12.6%
Smoking	51	9.8%
None of these	77	14.8%
Do you visit the doctor regularly?		
Yes	63	12.1%
No	459	87.9%

Table 3 shows population awareness regarding dry eye symptoms and risk factors in the Eastern region of Saudi Arabia. Three hundred and nine (59.2%) participants know what DES is and 285 (92.2%) think it is common in Saudi Arabia. Regarding causes of dry eye disease, the most reported were concentrating for extended periods while using electronic devices (410, 78.5%), climatic factors (dust, high temperatures, drought) (354, 67.8%), the lacrimal gland disorders (209, 40.0%), refractive surgeries (192, 36.6%), and medical conditions such as (diabetes, thyroid disorders, and rheumatoid arthritis) (190, 35.4%). As for symptoms, the most known symptoms were tingling and burning in the eyes (308, 59.0%), redness of the eye (305, 58.4%), eye strain (293, 56.1%), and foreign body sensation (274, 52.5%). Two hundred and twenty-six (43.3%) of the study participants reported scratching the surface of the cornea as one of the dry eye complications, followed by affected daily activities (213, 40.8%), eye inflammations and infection (207, 39.7%), and poor vision (194, 37.2%). Regarding preventive methods, the most reported included using moisturizing eyedrops (395, 75.7%), taking a break every 20 minutes when using electronic devices for extended periods (373, 71.5%), wearing sunglasses when going out (292, 55.9%), and avoiding going out in bad weather (246, 47.1%). 

**Table 3:** Population awareness regarding dry eye symptoms and risk factors in the Eastern region of Saudi Arabia

Awareness items	N	%
Do you know what dry eye disease is?		
Yes	309	59.2%
No	213	40.8%
Do you think that dry eye disease is frequent in Saudi Arabia? (n=309)		
Yes	285	92.2%
No	24	7.8%
Which of the following is a cause of dry eye disease?		
Concentration while using electronic devices for long periods	410	78.5%
Climatic factors (dust, high temperatures, drought)	354	67.8%
The lacrimal gland disorders	209	40.0%
Refractive surgeries	192	36.8%
Medical conditions such as (diabetes, thyroid disorders, and rheumatoid arthritis)	190	35.4%
I don't know	70	13.4%
Which of the following is a symptom of dry eye disease?		
Tingling and burning in the eyes	308	59.0%
Redness of the eye	305	58.4%
Eye strain	293	56.1%
Foreign body sensation	274	52.5%
Blurred vision	198	37.9%
Photosensitivity	183	35.1%
Secretions in or around the eyes	171	32.8%
Which of the following is a complication of dry eye disease?		
Corneal abrasions	226	43.3%
Impact on daily activities	213	40.8%
Inflammatory eye infection	207	39.7%
Poor vision	194	37.2%
I don't know	133	25.5%
Which of the following is a preventive measure of dry eye disease?		
Use moisturizing eye drops	395	75.7%
Take a break every 20 minutes when using electronic devices for extended periods	373	71.5%
Wear sunglasses when going out	292	55.9%
Avoid going out in bad weather	246	47.1%
place electronic devices below eye level	225	43.1%
Avoid smoking/setting near smokers	177	33.9%
I don't know	67	12.8%

Overall public awareness regarding dry eye diseases in Eastern region, Saudi Arabia. One hundred and forty-nine (28.5%) had an overall good awareness level, while 373 (71.5%) had poor awareness. Table 4 shows factors associated with public awareness regarding dry eye diseases in the Eastern region of Saudi Arabia. Eighty-two (40.2%) of participants aged 18-28 years had a good awareness of dry eye disease compared to 35 (19.3%) of others aged 36-55 with recorded statistical significance (p=0.001). Also, 56 (50.0%) of healthcare workers/students had overall good awareness versus 24 (17.8%) of non-employed participants (p=0.001). Moreover, 56 (50.0%) of healthcare workers/students had good awareness versus 93 (42.9%) non-employed/non-healthcare worker participants (p=0.001). Good awareness was detected among 33 (40.2%) of those who had undergone eye surgery compared to 116 (26.4%) of others (p=0.011). Also, 12 (50.0%) of those who used ointments to moisturize the eyes had good awareness versus 32 (22.5%) of those who did not use any method (p=0.001).

**Table 4:** Factors associated with public awareness regarding dry eye diseases in the Eastern region of Saudi Arabia P: Pearson X^2^ test; $: Exact probability test; * p < 0.05 (significant)

Factors	Overall awareness level				p-value
	Poor		Good		
	N	%	N	%	
Age in years					0.001*^$^
18-25	122	59.8%	82	40.2%	
26-35	91	77.8%	26	22.2%	
36-55	146	80.7%	35	19.3%	
> 55	14	70.0%	6	30.0%	
Gender					0.057
Male	120	66.3%	61	33.7%	
Female	253	74.2%	88	25.8%	
Job					0.001*
Not employed	111	82.2%	24	17.8%	
Health-care worker/student	56	50.0%	56	50.0%	
Non-health care worker/student	206	74.9%	69	25.1%	
Chronic diseases					0.591
Yes	99	69.7%	43	30.3%	
No	274	72.1%	106	27.9%	
Use of medications for Hypertension, pain relief, epilepsy, psychological diseases or Parkinsonism					0.651
Yes	93	69.9%	40	30.1%	
No	280	72.0%	109	28.0%	
Have you had eye surgery?					0.011*^$^
No	324	73.6%	116	26.4%	
Yes	49	59.8%	33	40.2%	
Do you use any of the following?					0.001*
Medical glasses	142	70.6%	59	29.4%	
Sunglasses	121	65.4%	64	34.6%	
Lenses	53	72.6%	20	27.4%	
Eye drops to moisturize	113	61.1%	72	38.9%	
Ointments to moisturize the eyes	12	50.0%	12	50.0%	
None of these	110	77.5%	32	22.5%	
Do you visit the doctor regularly?					0.232
Yes	41	65.1%	22	34.9%	
No	332	72.3%	127	27.7%	

## Discussion

DES refers to a group of tear film disorders due to reduced tear film production or instability, associated with ocular discomfort or visual symptoms and inflammatory disease of the ocular surface [2,8]. DES is a growing public health concern causing ocular discomfort, fatigue, and visual disturbance that interferes with quality of life, including aspects of physical, social, and psychological functioning, daily activities, and workplace productivity [9-10]. The current study aimed to assess awareness about dry eye symptoms and risk factors among the Eastern population in Saudi Arabia.

The study showed that more than one-fourth of the participants had a good awareness of dry eye diseases, manifestations and risk factors. In more detail, more than half of the participants know what DES is, and the majority of them, 285 (92.2%), expressed the belief that DES is prevalent in Saudi Arabia. Participants demonstrated a notable level of awareness regarding the causes of DES, with the most commonly reported factors including prolonged concentration while using electronic devices, climatic conditions, and lacrimal gland disorders. About one-third of the participants reported refractive surgeries and medical conditions such as diabetes, thyroid disorders, and rheumatoid arthritis. The most known symptoms were that more than half of the participants knew about tingling and burning in the eyes, redness of the eye, eye fatigue, and foreign body sensation. A lower awareness level was reported for DES-associated complications. Less than half of the study respondents knew about scratching the cornea's surface, followed by affected daily activities, eye inflammations and infection, and poor vision. On the other hand, awareness of the preventive measures was high as the use of moisturizing eye drops was reported among nearly three-fourths of the participants, followed by taking a break every 20 minutes when using electronic devices for extended periods. Wearing sunglasses when going out was known for over half of them, and avoiding going out in bad weather 246 (47.1%). A higher percentage of dry eye disease awareness was reported by Khalid et al. [7], where nearly half of the participants knew about the disease. Another study in Saudi Arabia showed that two-thirds of participants knew about dry eye disease [11]. Similar to the current study, A study in the Aseer region revealed that only 43 (8.7%) of participants had a good awareness level regarding dry eye disease, with an average knowledge score of 21.9 ± 9.0 out of 60 points [12]. In Jordan, Haddad et al. found that over half of the participants showed satisfactory awareness of DES [13].

The most common risk factor related to the disease was medication 226 (28.2%), followed by smoking 211 (26.3%) and diet 186 (23.2%). The current study showed that higher awareness was reported among young age participants who work in the medical care field and those who have undergone eye surgery. These and other risk factors were reported in the research [11-15]. The coronavirus disease 2019 (COVID-19) pandemic has also increased the need for screen use in daily life and at work. However, a number of factors have been linked to prolonged exposure to digital screens as the medical industry looks to increase its use of them for patient data storage and diagnostic imaging tests. As a result, it has been found that those who regularly use digital displays have a higher frequency of dry eye signs and symptoms [16]. Improved understanding of the causes of DES presents an opportunity to enhance disease diagnosis and management, as well as to develop more effective treatments for this prevalent and disabling condition. This knowledge can also guide public healthcare education programs and campaigns aimed at raising awareness about DES, highlighting its impact on daily life and the importance of seeking timely medical attention. By increasing public awareness about this condition, we can encourage individuals to take steps to manage their symptoms and seek treatment from healthcare providers [17]. This research has some limitations. First, the use of a self-reported questionnaire through social media can result in some bias while entering the data, as certain respondents might not answer all questions accurately. Second, a larger sample size will increase the accuracy and significance of this research due to smaller margins of error. Third, the lack of guidelines to assess the knowledge of the population regarding the disease. Finally, because there were not enough data collectors available in all of the eastern province cities during the data collection phase, the sample size was not distributed equally among the eastern cities.

## Conclusions

Participants showed unsatisfactory awareness regarding the causes of dry eye syndrome, where the most reported causes were prolonged concentration while using electronic devices, climatic factors, and lacrimal gland disorders. The knowledge of the dry eye and its contributing factors should be added to patient education and public dissemination. Further studies are needed to assess the awareness level in the eastern province of Saudi Arabia.
